# Cooperative pain education and self-management (COPES): study design and protocol of a randomized non-inferiority trial of an interactive voice response-based self-management intervention for chronic low back pain

**DOI:** 10.1186/s12891-016-0924-z

**Published:** 2016-02-16

**Authors:** Alicia A. Heapy, Diana M. Higgins, Kathryn M. LaChappelle, Joseph Kirlin, Joseph L. Goulet, Rebecca A. Czlapinski, Eugenia Buta, John D. Piette, Sarah L. Krein, Caroline R. Richardson, Robert D. Kerns

**Affiliations:** VA Connecticut Healthcare System, Pain Research, Informatics, Multimorbidities, and Education (PRIME) Health Services Research and Development Center of Innovation, 11ACLGS, 950 Campbell Avenue, West Haven, CT 06516 USA; Yale School of Medicine, New Haven, CT USA; VA Boston Healthcare System, Boston, MA USA; Boston University School of Medicine, Boston, MA USA; Widener University, Chester, PA USA; VA Ann Arbor Center for Clinical Management Research Health Services Research and Development Center of Innovation, Ann Arbor, MI USA; University of Michigan School of Public Health, Ann Arbor, MI USA; University of Michigan Medical School, Ann Arbor, MI USA

**Keywords:** Chronic low back pain, Clinical trial, IVR, Non-inferiority trial, CBT, Self-management

## Abstract

**Background:**

The Institute of Medicine report “Relieving Pain in America” recommends the promotion of patient self-management of pain for all people with pain. Given the high prevalence of chronic pain in the US, new strategies are needed to enhance access to cognitive behavioral therapy (CBT) and other evidence-based treatments designed to facilitate self-management of chronic pain conditions. Although CBT is efficacious, many patients have limited or no access to CBT. Technology-assisted delivery of CBT may improve access while maintaining efficacy.

**Methods/Design:**

We describe a randomized non-inferiority trial of interactive voice response (IVR)-based CBT for patients with chronic low back pain. This intervention uses daily IVR monitoring and weekly pre-recorded therapist feedback, based on patient-reported information, to provide treatment for patients at home. A total of 230 patients with chronic low back pain are being identified from a single statewide health system serving US military veterans. Participants are randomized to receive either ten weeks of in-person CBT or IVR-based CBT. The primary outcome is pain intensity as measured by the Numeric Rating Scale immediately post-treatment. Secondary outcomes include pain-related interference, emotional functioning, and quality of life measured immediately post treatment, and 6 and 9 months post recruitment. Exploratory objectives of the study are to examine: (1) potential mediators of impact on clinical outcomes (treatment retention, self-reported skill practice ratings, IVR call adherence, and treatment satisfaction); and (2) moderators of treatment engagement, adherence to therapist recommendations for pain coping skill practice, and effects on clinical outcomes.

**Discussion:**

This non-inferiority trial may identify an alternative to resource intensive in-person CBT that allows many more patients to receive care while also increasing retention of those enrolled in the program.

**Trial registration:**

ClinicalTrials.gov: NCT01025752. Registered 3 December 2009.

## Background

### Need for pain self-management

A recent Institute of Medicine (IOM) report estimates that approximately 100 million people in the US experience chronic pain at any one point in time [1]. Among the IOM committee’s recommendations is the widespread promotion of pain self-management for all people who experience pain. The report highlights the frequent barriers to care experienced by individuals with pain and encourages the development of strategies to address those barriers. Directly relevant to these two recommendations are investigations of novel ways to provide access to evidence-based self-management interventions. Although many interventions exist and individuals with pain may have a variety of preferences, cognitive behavioral therapy (CBT) is a mainstay of pain self-management interventions.

### Chronic pain and CBT

CBT has demonstrated efficacy for reducing pain and improving function in individuals with chronic pain. In a meta-analysis of psychological interventions for CLBP, Kerns and colleagues documented moderate to large effects of CBT and other psychological interventions in reducing pain and pain-related interference [2]. Similarly, a Cochrane review found combined cognitive and relaxation-based treatments were superior to a wait list control condition and resulted in moderate reductions in pain intensity [3].

The overarching goal of CBT is to assist the patient in developing an adaptive problem-solving, self-management approach to pain based on a conceptualization of pain as controllable and a personal attitude of self-efficacy and self-control. An important aspect of CBT is its foundation in a biopsychosocial and multidimensional perspective of chronic pain and the fact that CBT is specifically designed to target reductions in pain, disability, and emotional distress, while improving the patient’s overall quality of life. During therapy, a range of cognitive (e.g., attention diversion, development of coping self-statements) and behavioral (e.g., behavioral activation, activity pacing, relaxation) pain coping skills are taught. Patients are encouraged to practice the skills outside treatment sessions through assignment of specific goals for pain coping skill practice. Goal setting and coping skill practice are important components of CBT. Therapist reinforcement of pain coping skill practice, goal accomplishment, and problem solving, and support when goals are not successfully obtained are also important aspects of treatment.

Although CBT is an effective treatment for reducing pain and enhancing physical and emotional functioning, it has several features that have limited its availability in practice. A typical CBT treatment schedule may require weekly, 50-minute sessions for 6 to 12 weeks. This schedule can put treatment out of reach for patients with limited funds or transportation options, as well as for patients with health and mobility limitations or competing demands that do not easily accommodate weekly appointments. Trained therapists are often unavailable, especially in rural areas, low -income communities, or health systems that are far removed from academic medical centers [4]. As a consequence, CBT therapists only serve a small proportion of patients who might benefit [5]. As a partial solution to these access and cost barriers, new approaches look to mobile health technology, such as interactive voice response, as a way to increase the scale of sustainable programs for patients without the need for 1–1 in-person encounters.

### Interactive Voice Response (IVR)

IVR is an automated telephonic technology that allows patients to report and receive information via their mobile or landline telephone. Calls can be placed at times that are convenient to the patient, and data are collected when patients answer pre-recorded voice prompts using their telephone key pad or voice. Patients also can receive information via IVR such as pre-recorded didactic information regarding pain coping skills or personalized therapist feedback. The therapist is able to monitor a patient’s daily IVR reports and tailor subsequent feedback based on pain-related symptoms, treatment engagement or treatment adherence.

### Use of IVR to deliver treatment

There is emerging evidence that IVR-based interventions are effective for providing education, peer support, and tailored messages to enhance adherence and maintain treatment gains for patients with chronic health conditions [6–10]. Reviews of the broader literature of technology-assisted interventions beyond IVR for conditions like depression and anxiety have concluded that, although technology-assisted interventions are efficacious, some form of therapist contact enhances not just treatment effects, but also treatment retention [11–13]. Within the realm of pain-related research, Naylor and colleagues found that the use of IVR after in-person CBT to reinforce the use of pain coping skills can maintain and even enhance gains made in treatment, reducing the use of opioid-based pharmacotherapy [8, 9, 14]. Also, studies have shown high levels of adherence to daily IVR telephone calls whether participants received payments for calls or not [6, 14]. Despite these promising results, to our knowledge there have been no trials of a solely IVR-based treatment for chronic pain.

### Conceptual framework

#### Non-inferiority trial of IVR-based CBT for chronic pain

When a new treatment promises potential benefits such as improved access, fewer side effects, or lower cost relative to an established treatment, but there is no hypothesized reason to believe that the new treatment is more efficacious, a non-inferiority trial can be used to examine the relative benefits and efficacy of the two alternatives. A new treatment is considered to be non-inferior based on evidence that the decrement in efficacy of the new treatment relative to the established treatment, by a pre-specified margin (called the “non-inferiority margin”) [15]. For example, in a non-inferiority trial of pain management treatments such as the one conducted, if the actual difference in mean pain intensity between IVR-based CBT (IVR-CBT) and in-person CBT is less than the pre-defined non-inferiority margin, IVR-CBT will be judged to be non-inferior to in-person CBT. The primary rationale for this trial is that IVR-CBT offers potential benefits (e.g., improved access, lower patient burden) relative to in-person CBT, wherein the tradeoff of a slight decrement in efficacy can be tolerated in return for enhanced access to care. If IVR-CBT is found to have clinically non-inferior outcomes relative to in-person CBT, it will be recommended for use to patients with chronic low back pain (CLBP) and could be adopted as an alternative to the current, more costly approach.

An important objective in translating CBT to the IVR environment was to promote practice of the pain coping skills through goal setting while retaining the therapist contact and reinforcement that is associated with positive patient outcomes. We sought to leverage the ability of the IVR system to provide brief, automated contact with participants to not only assess skill practice adherence, but to provide a sense of regular contact and to enhance feedback.

### Key intervention components

#### A pedometer-assisted graduated walking program

Participants in both treatment conditions are given a pedometer to facilitate their engagement in the walking component of the treatments. The walking program is progressive and participants are assigned a weekly goal of increasing their average daily step count by 10 % over the prior week’s average step count beginning in week three when the physical activity module is presented in both the IVR and “live” therapist arms of the trial.

#### Patient handbooks

At baseline, participants in both conditions receive handbooks that describe the CBT treatment skills and weekly goals, and contain instructions for using the IVR system and pedometer. For patients randomized to IVR-CBT the handbook information is also made available during IVR calls. Easy accessibility of this information is critical for these patients because they cannot rely on the therapist for its presentation and reinforcement. In the in-person condition, information about pain coping skills is conveyed primarily through the therapist during weekly sessions and the companion handbook contains shortened versions of the pain coping skill explanations contained in the IVR-CBT handbook.

#### Skill practice and meaningful activity goals

Each week, participants are assigned a daily skill practice goal that corresponds to the specific pain coping skill presented in treatment that week (e.g., Week 4: practice deep breathing for 5 minutes each day). The *skill practice goal* for each week is described in the patient handbook for participants randomized to the IVR-CBT condition and is assigned by the therapist during the weekly treatment session for participants randomized to the in-person CBT condition. Participants in both conditions are assigned the same skill practice goals (see Table 1). As participants progress through treatment, they are encouraged to continue practicing skills learned in prior modules. Participants also create their own *goal for engaging in pleasant or meaningful activities* to promote behavioral activation (i.e., planned rewarding or productive activities in order to enhance mood and activity levels). Participants in the IVR-CBT condition are guided in this effort by information in the patient handbook. Participants in the in-person CBT condition collaborate in setting goals with their therapist during treatment sessions.

**Table 1:** Treatment modules and goals for CBT and IVR-CBT

Week	Coping Skill	Description	Goals
0	None	Baseline assessment	None
1	Introduction	Present rationale for treatment, explain pain cycle and introduce goal setting.	- Complete one exercise each day in the “Your Guide to Setting Meaningful Activity Goals” section
2	Stretching	Introduce stretching, its benefits, explain acute vs. chronic pain, beliefs about pain and provide suggested stretches to practice.	-Practice stretches provided in the handbook daily
			-Set/ work on meaningful activity goal
3	Movement, Walking & Body Mechanics	Instructions for walking, body mechanics, increasing activity and preventing injuries.	-Practice body mechanics
			-Increase daily steps +10 % of prior week’s steps
			- Set/ work on Meaningful activity goal
			-Continue to practice prior week’s skill
4	Deep Breathing	Instructions for diaphragmatic breathing and its benefits.	-Practice diaphragmatic breathing using CD for 5–10 minutes/day
			-Increase daily steps +10 % of prior week’s steps
			- Set/ work on meaningful activity goal
			-Continue to practice prior weeks’ skills
5	Progressive Muscle Relaxation	Instructions for progressive muscle relaxation and its benefits.	-Practice progressive muscle relaxation using CD
			-Increase daily steps +10 % of prior week’s steps
			- Set/work on meaningful activity goal
			-Continue to practice prior weeks’ skills
6	Identifying Unhealthy Thoughts	Influence of negative thoughts on pain, activities and mood.	-Practice catching unhealthy thoughts
			-Increase daily steps +10 % of prior week’s steps
			- Set/work on meaningful activity goal
			-Continue to practice prior weeks’ skills
7	Balancing Unhealthy Thoughts	Instructions for challenging and changing negative thoughts	-Practice catching and changing unhealthy thoughts
			-Increase daily steps +10 % of prior week’s steps
			- Set/work on meaningful activity goal
			-Continue to practice prior weeks’ skills
8	Time-Based Pacing	Instructions for time-based pacing and its benefits.	-Practice time-based pacing by applying it to one activity daily
			-Increase daily steps +10 % of prior week’s steps
			- Set/work on meaningful activity goal
			-Continue to practice prior weeks’ skills
9	Sleep Hygiene	Common sleep problems and sleep hygiene tips.	-Practice at least one sleep hygiene tip daily
			-Increase daily steps +10 % of prior week’s steps
			- Set/ work on meaningful activity goal
			-Continue to practice prior weeks’ skills
10	Planning for the Future	Relapse prevention; reviewing goals and accomplishments, managing relapse and pain flare ups.	- Complete 1 section of the pain flare prevention handout daily.
			-Increase daily steps +10 % of prior week’s steps
			- Set/work on meaningful activity goal
			-Continue to practice prior weeks’ skills

#### IVR assessment

Participants in both conditions receive daily automated calls from the IVR system. Calls are scheduled to occur each evening at the same time within the daily assessment window of 6:00 PM-10:00 PM in order to ensure that each call represents a 24-hour reporting period. Calls are scheduled at a time of the participant’s choosing. Participants are also able to connect directly with the Department of Veterans Affairs (VA) Veteran Crisis Line. The crisis line is staffed 24-hour a day and offers callers (or those who use confidential chat or text) support, crisis response, and facilitates follow-up in-person care at a veteran’s local VA medical center.

#### Weekly therapist feedback

Participants in the IVR-CBT condition receive a weekly, two to five minute pre-recorded personalized message from their therapist via the IVR system. On the last day of each week participants are told that they have a message from their therapist. This message may be accessed and replayed as often as the participants want. If participants miss the call that contains the feedback message, they are prompted to listen to the message during their next call. Participants and therapists may record messages to one another on the IVR system at any time during treatment. This allows participants and therapists to obtain clarification or feedback on specific topics and avoid frustration that may impair progress or prompt dropout.

## Methods/Design

**Fig. 1:**
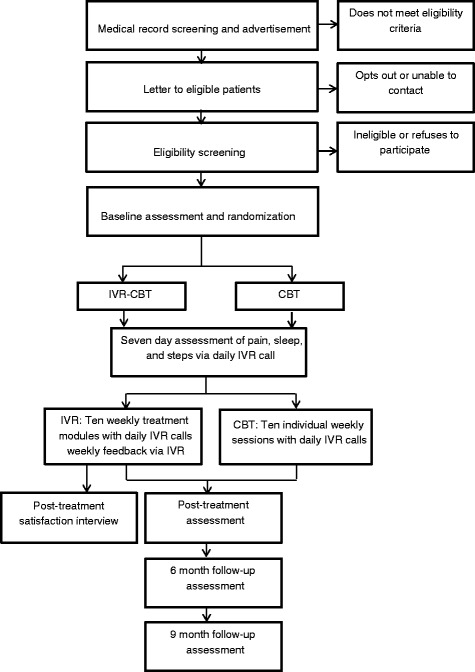
Study recruitment, enrollment, treatment, and assessment processes

This randomized controlled non-inferiority trial includes assessment of outcomes at baseline, post-treatment (i.e., 3 months), 6 months and 9 months (See Fig. 1) with post-treatment being the primary timepoint. The aims of the study are:

- Aim1: To determine whether IVR- CBT for chronic low back pain is non-inferior or “not unacceptably worse” [15] than the gold standard in-person treatment. The primary outcome is pain intensity as measured at post-treatment by the 11-point [0 (no pain) to 10 (worst pain imaginable)] Numeric Rating Scale (NRS) for pain. The non-inferiority margin was set at one point on the NRS.
- Aim 2: To determine whether IVR-CBT enhances participants’ physical and emotional functioning, and health-related quality of life immediately post-treatment and each follow-up relative to in-person treatment.
- Aim 3: To determine whether treatment groups differ with respect to important mediators of outcomes, including treatment dropout rates, self-reported skill practice ratings, IVR call adherence, and treatment satisfaction.
- Aim 4: To identify key moderators of improvements in treatment outcomes including age, sex, race/ethnicity, number of pain sites and location, medication use*,* and psychiatric comorbidities.

Eligible patients are randomized to receive either standard in-person CBT for CLBP or IVR-CBT. This study was approved by Institutional Review Boards at Yale University School of Medicine and the VA Connecticut Healthcare System (VACHS).

### Eligibility

#### Experimental subjects and controls

Participants are 230 patients with CLBP receiving treatment at the VACHS. Specific eligibility criteria include: 1) presence of at least a moderate level of pain (i.e., pain intensity rating of ≥ 4 on the 0 to 10 NRS) for a period of ≥ 3 months; 2) diagnosis of a low back pain condition in the electronic health record (EHR); 3) absence of a medical or psychiatric condition that could impair participation (e.g., severe COPD, terminal cancer, active substance abuse, psychosis, suicidality or severe depression Beck Depression Inventory-II score >28 ); 4) self-reported ability to walk at least one block without chest pain; 5) absence of dementia defined by St. Louis University Mental Status examination (SLUMS); [16] 6) absence of surgical interventions for pain during participation in this study (participants undergoing surgery will be discharged from the study in order to maintain the integrity of the active treatments); 7) availability of a touch-tone landline or mobile telephone; and 8) absence of any sensory deficits that would impair participation (e.g., hearing loss to a degree that telephone usage is not possible).

#### Recruitment

Participants are recruited from the VACHS by mailing letters to those who are identified via EHR review as having a back-pain-related diagnosis including low back and spine conditions and nerve compression (ICD-9 codes 724.02, 724.03, 724.2, 724.3, and 724.4) and a pain intensity rating of ≥ 4 (indicating moderate pain) [17] on the 0–10 NRS during their most recent clinic visit. Potential participants are called to solicit enrollment unless an “opt out” response is received by study staff, either by returning a pre-stamped and addressed letter or by telephone call. Additional participants are obtained through provider referral and advertisements placed in clinical care areas throughout the medical center and outpatient clinic locations of VACHS. Potential participants who respond to the referral or advertisement are screened by the study research assistant regarding key eligibility criteria for the study (e.g., confirmation of presence of chronic back pain of at least moderate intensity and absence of medical and psychiatric comorbidities that preclude eligibility). If initial screening suggests potential eligibility, a face-to-face appointment is scheduled for obtaining written informed consent and a full eligibility screening.

#### Baseline appointment

After consent, the final assessment of study eligibility is determined through review of the EHR by the study psychiatric advance practice registered nurse and psychologists, and participant responses to validated measures. The study nurse verifies the diagnosis of CLBP and uses a structured chart abstraction and assessment tool based on a classification system recommended in clinical practice guidelines [18] to identify the diagnosis and treatments of CLBP and classify participants’ pain as either non-specific low back pain, low back pain with a radicular component, or pain associated with other specific spinal causes. A trained research assistant under supervision of a psychologist reviews each participant’s chart to identify disqualifying psychiatric diagnoses or active suicidality. The research assistant administers a brief semi-structured interview to collect demographic and pain-related information, the Mini-International Neuropsychiatric Interview for DSM-IV (MINI) [19] to identify the presence of any DSM-IV psychiatric disorders, the Beck Depression Inventory-II (BDI-II) [20] to screen for severe depressive symptoms, and the SLUMS examination to screen for cognitive deficits likely to interfere with treatment participation. Prescribed pain medications and their dosage, as noted in the EHR, are recorded. Individuals who meet eligibility criteria are enrolled in the IVR system and TrialDB, a secure, encrypted clinical trials management database that facilitates participant randomization and collection and management of questionnaire data.

#### Randomization

Eligible participants are randomized to either in-person CBT or IVR-CBT immediately after completing the baseline assessments. The study research assistant enters the participant’s demographic information and stratification factors into TrialDB, which holds the randomization schedule and is masked from users thereby enabling concealed allocation. The treatment allocation ratio is 1:1 using a permuted stratified block design with variable block size. Randomization is done within strata defined by patients’ distance from the VACHS-West Haven campus (<10, 11–25, 26 or greater miles) and cause of low back pain as determined by the study nurse based on EHR review (non-specific low back pain, low back pain with a radicular component, or pain associated with other specific spinal causes) [18]. The research assistant informs a study therapist of the participant’s treatment assignment.

#### Pedometer, TrialDB, and IVR system training

The research assistant enrolls participants in the IVR system and TrialDB, a separate, secure, encrypted web-interface used for completing the study outcome measures. Participants are provided with training in the use of TrialDB, the IVR system and the study pedometer by a brief demonstration supplemented by written instructions included in the patient handbook. Participants’ stride length is measured and they are provided with an Omron Go Smart Model HJ-112 pocket pedometer to use in the walking portion of the CBT and IVR-CBT interventions.

#### Patient handbooks

At the end of the baseline appointment participants are given either a CBT or IVR-CBT patient handbook.The patient handbooks were adapted from materials used in a prior trial of in-person CBT for CLBP by psychologists with experience in the delivery of CBT for chronic pain [21]. The materials are written at a 6^th^ grade reading level and pictures and simple figures are incorporated as often as possible to enhance patients’ understanding and engagement. As a check on comprehension, participants are asked five true/false questions about each treatment module in the week it is presented. The IVR-CBT patient handbook and IVR scripts were pilot tested with 17 individuals with chronic pain who reviewed the materials and an additional four individuals who received treatment using the new materials. Both groups provided feedback via semi-structured interviews and revisions were made to the materials based on feedback.

#### Daily IVR calls

After enrollment, but prior to beginning treatment, participants receive seven daily IVR calls to assess their baseline pain intensity, pedometer-measured step counts and self-reported sleep duration in hours [22]. Starting on the first day of treatment and continuing for 70 days, participants receive daily IVR calls to answer seven daily questions that assess pain intensity, sleep quality and duration, pedometer-measured step count, catastrophizing (I worried my pain would never end”, “I felt my pain was so bad I could not stand it anymore”) and adherence to the current week’s skill practice goal. Once per week participants are asked to report: 1) any adverse events associated with the graduated walking portion of the treatment, 2) any increase or decrease in pain medication dose made on the advice of their physician or their own initiative, 3) how often they practiced their weekly, self-selected pleasant or meaningful activity goal and if it improved their happiness or satisfaction 4) if they continued to use any of the pain coping skills learned in prior weeks, and 5) their comprehension of the module material via five true/false questions about the week’s pain coping skill. All of the information reported during a call is automatically captured in a database and time and date stamped for later review by a therapist. Participants receive a call at their chosen time; if they do not complete the call, they are called again fifteen minutes after the designated time, and if they do not complete the second call, they are called again one hour later. If they do not answer any of these calls, the call day is considered missed and they are not called again until the following day. Participants have the option of calling into the system to make their daily report in circumstances when they are unavailable at the scheduled call time. The IVR system flags any instances of a participant missing the first call day or two consecutive call days, and the study research assistants contact the participant to assist them if there is a technical difficulty or to encourage adherence to the call schedule. When participants are unable to complete calls for an extended period of time such as during vacation, calls can be paused and resume when a participant returns. Participants are not paid for completing daily IVR calls.

### Treatments

#### Overview

Both CBT conditions include 10 treatment modules delivered over 10 consecutive weeks. Treatments are delivered by either a PhD-level psychologist or the study nurse trained and supervised by a clinical psychologist with specific competencies and experience in delivering CBT for chronic pain. Therapists follow treatment manuals and participants are provided with a treatment-specific participant handbook. The 10-week course of therapy consists of an introductory module, followed by 8 consecutive pain coping skills modules, and concludes with a 10th module emphasizing skill consolidation and relapse prevention. Both conditions include the same pain coping skills, which were selected from a wider collection of possible skills because they were rated most highly by participants in a prior funded trial on importance, interest and ability to engage in them [21] (see Table 1 for description of skills). Participants assigned to both conditions continue to receive routine care of their CLBP by their current healthcare providers. Study staff do not attempt to influence clinical care other than CBT in any way.

*CBT* involves weekly, 30-minute, individual in-person sessions with a therapist, a pedometer-facilitated walking program and daily IVR assessment. Each session includes one treatment module.

Each module includes: (1) the presentation of an explicit rationale for development and use of the specific coping skill being taught, (2) a description of each coping skill, skill modeling and practice of the skill during the session, (3) problem-solving about the practice and use of the specific skill, and (4) assignment of a specific goal for practice of the week’s skill and collaboration between the therapist and participants to develop a weekly goal for engaging in pleasant or meaningful activities.

*IVR-CBT* is an adapted form of in-person CBT specifically designed for the IVR environment. IVR-CBT involves reading handbook materials, a pedometer-facilitated walking program, daily IVR assessment and retrieval of pre-recorded therapist feedback related to treatment engagement and goal completion. Participants in the IVR-CBT condition have access to extra IVR features not available to participants in the in-person CBT condition. For example, optional pre-recorded, brief audio segments are provided to reinforce the information about pain coping skills that are presented in the patient handbook and offer participants with poor literacy skills an additional opportunity to learn the treatment skills. Peer testimonials feature a voice actor speaking the words of an actual participant in one of our prior trials of CBT for chronic pain describing their successful use of the pain coping skills. A collection of short motivational tips also are available.

#### IVR-CBT therapist feedback

**Fig. 2:**
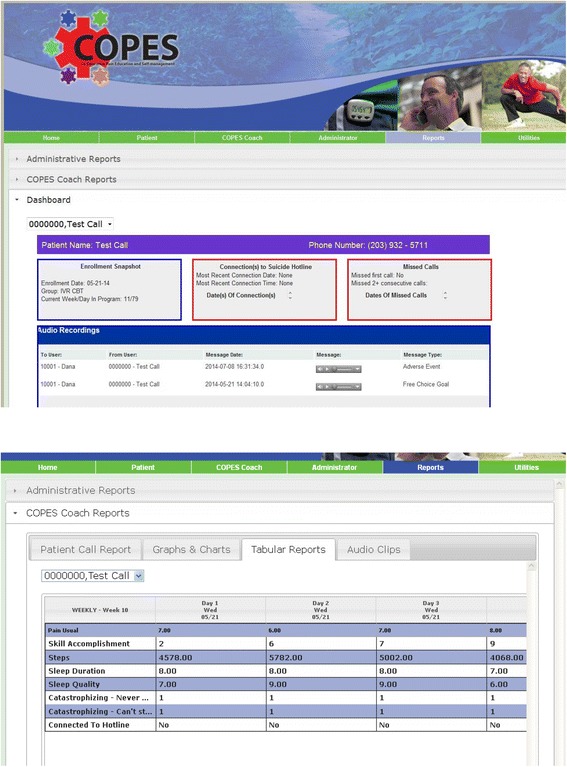
Screen shots of study dashboard views

Each week the therapist reviews participants’ IVR-reported data via a web interface that summarizes the daily reports (see Fig. 2). Therapists comment on participants’ average pain intensity, sleep, steps, and skill practice for the week and note the weekly average, any change from the prior week, either positive or negative, and note any patterns (e.g., more pain on weekends versus week days). Therapists also provide reinforcement for any goal accomplishment and note potential associations among pain, goal accomplishment, and pain coping skill practice. Therapists provide corrective feedback on any incorrect answers to the true/false questions. Finally, therapists assign a steps goal for the upcoming week.

#### Treatment fidelity measures

In-person CBT and IVR-CBT Treatment Receipt/Comprehension: The measures of treatment receipt (i.e., the five- item true/false content questionnaire) have been described above. We will analyze and report these data regarding average level of skill comprehension and whether any treatment modules were more difficult for participants to understand. Treatment fidelity will be assessed by psychologists with expertise in CBT for chronic pain, who will rate audiotapes of 30 % of the in-person CBT sessions and the IVR-CBT weekly feedback recordings to assure that key components of the manuals are covered. Percentages of treatment integrity/violations will be calculated. The study’s psychologist principal investigator will provide corrective feedback to the psychologist whenever drift occurs.

#### Adverse events

Although walking is not considered risky for patients with chronic musculoskeletal pain and is often recommended, information about adverse events associated with participation in the walking portion of the treatment is solicited once per week during the IVR assessment calls. Participants may report adverse events by recording a brief audio message. These messages are reviewed each business day by study staff and any reports that indicate a cause for concern are followed-up with the participant, their primary care provider, or both.

#### Outcomes

Survey outcomes are assessed at 4 time points, baseline, post-treatment, and 6 and 9 months post baseline. Participants are given up to 14 weeks to complete treatment in order to accommodate rescheduled sessions due to vacations, illness or travel difficulties. Post-treatment data are collected when a participant completes treatment or at 15 weeks post-baseline regardless of whether the participant has completed treatment. Survey outcomes are assessed using the TrialDB system, which allows participants to complete the self-report questionnaires online via a secure web-based interface. Participants who do not have an Internet connection may come to the medical center to complete the measures via TrialDB or complete a paper version of the measures and return them by mail. IVR outcomes will be assessed daily during the baseline week and over the 10 treatment weeks.

Measures were selected based on the Initiative on Methods, Measurement and Pain Assessment in Clinical Trials (IMMPACT) recommendations [23], which called for the assessment of multiple domains of the pain experience in all pain treatment trials and recommended measures based on their psychometric properties and respondent burden. These measures are consistent with CONSORT guidelines for non-inferiority trials [24] that recommend that outcome measures be similar to those used in studies to establish efficacy of the reference treatment.

*Primary Outcome - Pain intensity:* We assess participants’ average pain intensity over the past week via survey, at the four outcome assessment points using the Numeric Rating Scale of pain intensity (NRS-I) [25] an 11-point numeric rating scale (0 = no pain, 10 = worst pain imaginable). The primary outcome is the average weekly pain intensity at post-treatment.

*Secondary Outcomes – Pain intensity* will also be assessed daily during the trial via the IVR system using the NRS scale. Subjects will have a maximum of 77 daily scores: 7 daily calls for the baseline assessment week and 7 daily calls for each of the 10 weeks of treatment. We will use these scores to compute weekly means for each subject. The mean of seven daily assessments has been found to provide reliability in excess of 0.90 [26].

*Physical functioning:* The nine-item Interference subscale of the West Haven-Yale Multidimensional Pain Inventory (WHYMPI) is used to assess pain-related interference in social, work and household activities and has demonstrated good internal consistency (.86) and stability (.85 over 2 weeks) [27]. The 24 item Roland and Morris Disability Questionnaire is designed to assess physical functioning in patients with low back pain [28]. It has demonstrated good internal consistency (.84-.93) and stability (.83 over 3 weeks) [29].

*Emotional functioning:* Overall emotional functioning is assessed using the 65-item Profile of Mood States (POMS) [30], a multidimensional measure of emotional functioning designed to assess six dimensions of mood and to be used in non-psychiatric or physically ill populations. Internal consistency (0.84 to 0.95, depending on subscale) and test-retest reliability (0.65 to 0.74, depending on subscale) is good. Depression symptom severity is assessed using the 21-item Beck Depression Inventory-II [31] (BDI-II) a widely used self-report measure with excellent internal consistency (.94) and evidence of convergent and discriminant validity in primary care medical patients (.73–.96) [20].

*Quality of Life*: The Veterans SF-36 is used to assess health-related quality of life. This measure has demonstrated good internal consistency (.78–.93 across 8 subscales) and is strongly correlated with socioeconomic status and morbidities [32].

### Tertiary outcomes

*Adherence to coping skill practice* is assessed via IVR. Participants rate their daily practice of each week’s skills on a 0 (not at all accomplished) to 10 (completely accomplished) scale for each of the specified goals.

#### IVR call adherence

Call adherence is calculated as the total number of IVR calls made divided by the total number of expected calls (77).

#### Patient satisfaction

Patient satisfaction is assessed by a modified version of the Client Satisfaction Questionnaire-8 [33], an 8-item satisfaction survey designed to assess global satisfaction with treatment. This measure has been used widely to assess treatment satisfaction across numerous types of interventions and demonstrates good internal consistency (α = 0.87–0.93 across three samples) and has shown correlation to treatment attendance and retention.

#### Treatment attendance and drop out

Session attendance is tracked and the number of participants who do not complete follow-up assessments is calculated. In order to understand reasons for treatment dropout and evaluate “missingness” we prospectively collect data regarding reasons for treatment dropout and missing data. The research assistant, who monitors IVR and questionnaire completion, collects these data.

#### Sample size calculation

The sample size was calculated based on a test of non-inferiority comparing IVR-CBT to CBT, with 80 % power, Type I error (one-sided) of 0.025, and assuming the true difference between means is 0. Based on preliminary data from similar participants in an ongoing study of the efficacy of CBT for chronic back pain [21], the estimated baseline NRS pain intensity score will be 7 ± 2.45 units. A 20 % reduction in the NRS pain score from baseline to post-treatment, that is, from 7 to 5.6 or 1.4 units, is considered to be clinically relevant. The non-inferiority margin was set at one NRS unit, so that if the mean pain rating for IVR-CBT is less than one unit higher than the mean in-person CBT score (mean IVR-CBT < mean in-person CBT + 1), IVR-CBT will be deemed non-inferior. Conducting a test of this hypothesis can be done by comparing the upper limit of the 95 % confidence interval for the mean pain difference between IVR-CBT and in-person CBT with the non-inferiority margin 1; if this upper limit falls below 1, we will be able to conclude IVR-CBT is non-inferior. Based on these assumptions and 15 % inflation for losses, the total required sample size is 230 participants (115 per group).

### Analysis plan

#### Baseline analysis

The adequacy of the randomization will be assessed by comparing baseline demographic and clinical characteristics between the two treatment groups. Additional covariates will include number and location of pain sites and difference in medication use at baseline in order to assess whether these variables are associated with a differential response to the treatment.

#### Analysis

We present below the analysis of primary and secondary outcome measures only. Analysis of primary and secondary outcomes will employ linear mixed-effects models for longitudinal data, which will account for the clustering induced by repeated measures on individual patients. Mixed effects models make use of all available measurements on subjects and provide valid inferences even in the presence of missing data as long as the missingness is at random. Data from different subjects are assumed to be independent, while the correlation structure of the repeated measurements within subjects is modeled via parameterization of the covariance structure.

Between-group comparisons of the effectiveness of IVR-CBT and in-person CBT at each assessment time point will be conducted. Each of these comparisons can be tested separately within the same mixed-effect model using a treatment dummy variable, time dummy variables, and treatment-by-time interaction terms and appropriate contrasts. The outcome variable in each model will be the outcome at the follow-up time points, with the baseline value of the outcome and the stratification variables included as covariates in the model. Results will be summarized as LS- means (and their 95 % confidence intervals) within and between treatment groups at each time point. Non-inferiority will be demonstrated by the upper limit of the 95 % confidence interval for the LS-mean pain difference between IVR-CBT and in-person CBT *at post-treatment* being less than 1. Following CONSORT recommendations [24], non-inferiority analyses will be conducted both on a *per protocol* (e.g., having completed 3 weeks of treatment which is considered a dose of treatment) basis and an *intent-to-treat* basis. All other analyses will be conducted according to intent-to-treat basis, that is, by considering patient group status as randomized.

#### Missing data

We will compare the distribution of lost patients by reason between the two study groups to evaluate any differences in the reasons for losses. We will compare the rate of loss to follow-up between the two groups using the chi-square test, and if this rate is different between the two groups, interpretation of the results will be made in view of this finding. If we find any baseline variables to be associated with the loss to follow-up, then we will include these baseline variables as covariates in the models evaluating the intervention effect. In case of problematic missingness, we will use multiple imputation based on sequential regression imputation to impute missing variables. Following Rubin's method [34] for multiple imputation inference, each of the simulated complete datasets will be analyzed by standard methods, and the results will be combined to produce estimates and confidence intervals that incorporate missing-data uncertainty.

## Discussion

To our knowledge, this study represents the first trial of CBT for chronic pain that uses IVR as the sole means to deliver a pain self-management intervention. Prior studies have used IVR, but only to maintain treatment effects achieved via “live” therapy. If shown to be non-inferior to in-person CBT in terms of efficacy, IVR-CBT could provide an avenue for accessing empirically validated psychological treatment for chronic pain to those who are unable or unwilling to attend in-person treatment. Another innovative aspect of this trial is the use of a non-inferiority design that allows us to directly compare the IVR-based treatment to the gold standard, in-person CBT. Despite the recent increase in the development and testing of technology-assisted interventions for chronic pain and other common conditions, these treatments are seldom formally compared to the in-person treatment they seek to replace.

In addition to enhancing access to treatment, IVR may offer advantages as a data collection method for measurement of patient-reported outcomes and examining the process of behavior change. During in-person treatment, assessment of pain intensity, pain-related interference, emotional functioning, and adherence to goals set in prior sessions occurs during the patient’s therapy sessions, often not immediately proximal to its occurrence, making the reports retrospective in nature. Retrospective patient reporting, often using pencil and paper methods, is the most common technique for collecting information regarding a person’s pain experience in both clinical research and treatment. Despite the popularity and ease of use of retrospective self-reports, this method is vulnerable to recall and cognitive biases that attenuate their validity and reliability [35, 36]. Collecting data via a daily IVR call allows the data to be collected prospectively, removing many of the limitations of retrospective reports.

IVR-based treatment methods facilitate the collection of daily patient-reported outcomes, which enable the examination of treatment processes. Little is known about the process by which people change their behavior and obtain treatment benefits, and commonly used analytic methods do not allow the fine grained depiction of the process of change between pre- and post-treatment, though there have been some investigations [37, 38]. Most existing models assume that the rate of change is constant over time, but outcomes and predictors may change over the course of treatment. In this study the collection of daily data over the course of 11 weeks provides high-frequency longitudinal data. These longitudinal data are appropriate for analytic methods like time varying effect models that allow us to examine change over time in both outcomes and predictors. These multiple assessment points enhance our ability to detect the shape of the curve associated with change and when in the treatment process change takes place (is it constant or does change occur primarily at a specific time during treatment). Thus variables that are thought to affect pain intensity like activity level, sleep, catastrophizing and skill practice are not assumed to be constant over the course of treatment, but are allowed to vary over time. This will allow us to determine the change over time in a number of factors thought to have an impact on pain intensity and compare the process of change in treatment responders (those who obtain at least a 30 % reduction in pain intensity at post-treatment) and non-responders.

CLBP is one of the most prevalent and costly healthcare problems in industrialized nations and a common reason for healthcare use [1, 39]. Given the large number of individuals who could potentially benefit from empirically validated treatments such as CBT for chronic pain, it is important that barriers to this treatment are addressed. An IVR-based CBT approach can be used to enhance care for patients when face to face access is not feasible.

## Abbreviations

APRN: advance practice registered nurse
BDI-II: beck depression inventory-II
CBT: cognitive behavioral therapy
CLBP: chronic low back pain
EHR: electronic health record
DSM-IV: diagnostic and statistical manual of mental disorder – version 4
IMMPACT: initiative on methods, measurement and pain assessment in clinical trials
IOM: Institute of medicine
IVR: interactive voice response
IVR-CBT: interactive voice response-based cognitive behavioral therapy
MINI: Mini-International Neuropsychiatric Interview for DSM-IV
NRS: numeric rating scale
POMS: profile of mood status
RMDQ: Roland Morris disability questionnaire
SLUMS: Saint Louis University Mental Status Examination
VA: Department of Veterans Affairs
VACHS: VA Connecticut Healthcare System
